# Antimicrobial resistance and plasmid-associated virulence genes in* Salmonella* isolated from pigs, pork, and humans in border provinces of Thailand and neighboring countries

**DOI:** 10.7717/peerj.19884

**Published:** 2025-08-25

**Authors:** Siraphatson Wetchasirigul, Jiratchaya Puangseree, Sunpetch Angkititrakul, Rangsiya Prathan, Songsak Srisanga, Rungtip Chuanchuen

**Affiliations:** 1 Research Unit in Microbial Food Safety and Antimicrobial Resistance, Department of Veterinary Public Health, Faculty of Veterinary Science, Chulalongkorn University, Bangkok, Thailand; 2 Research Group for the Prevention Technology in Livestock, Faculty of Veterinary Medicine, Khon Kaen University, Khon Kaen, Thailand; 3 Center for Antimicrobial Resistance Monitoring in Food-borne Pathogens, Faculty of Veterinary Science, Chulalongkorn University, Bangkok, Thailand

**Keywords:** R plasmids, Virulence plasmid, *Salmonella*, Thailand

## Abstract

In Southeast Asia, most antimicrobial resistance (AMR) data on *Salmonella* have been generated at the phenotypic level, while insights into the genetic characteristics of AMR and virulence genes remain limited. This study aimed to further characterize AMR *Salmonella* isolates carrying plasmid-associated virulence genes in Thailand and neighboring countries. A total of 366 *Salmonella* isolates were collected from pigs (*n* = 265), pork (*n* = 69), and humans (*n* = 32) across Thailand, Lao People’s Democratic Republic, Cambodia, and Myanmar. Overall, 69.40% (*n* = 254/366) were multidrug resistant, including resistance to medically important antimicrobials tigecycline, azithromycin, colistin and ciprofloxacin. Whole genome sequencing analysis revealed that five *Salmonella* Enteritidis ST11 clinical isolates from different patients in different provinces carried IncFIB/IncFII plasmids with plasmid-associated virulence genes (*spvBCD, pefABCD*, *rck*, and *mig-5*), of which three of them (SA615, SA616 and SA617) additionally harbored IncX1 plasmid carrying *bla*_TEM-135_. Virulence plasmids (pSEVTs) exhibited a close relationship with the pSLT of *Salmonella* Typhimurium LT2 except for two absent segments (PSLT056-PSLT057-PSLT059 PSLT060-PSLT061-PSLT062-PSLT063-064-ssB-PSLT067) and (*traR-traC-trabI-traW-traU trbC-traN-trbE-traF-traQ-traQ-trbB-traH-traD-traH-traI-traX*) as well as two invert regions, R1 (locus tag PSLT001 to *repA2*) and R2 (PSLT025 to *finO* with deleted *tra*) in pSEVTs. None of the plasmids were horizontally transferred under ampicillin selective pressure. Phylogenetic analysis of whole genome sequence and virulence plasmids revealed the clonal dissemination of the isolates. The co-existence of virulence and resistance plasmids may complicate antibiotic therapy, highlighting the need to monitor plasmid-associated virulence genes alongside AMR genes in surveillance programs for humans and animals.

## Introduction

As a global health emergency of the 21st century, antimicrobial resistance (AMR) disproportionately impacts low- and middle-income countries (LMICs) due to poverty, inequality and poor basic sanitation (Antimicrobial Resistance Collaborators, 2022). Southeast Asia is a recognized global hotspot for the emergence and spread of AMR, with AMR and multidrug resistance (MDR) consistently documented in various foodborne pathogens (Nhung et al., 2016). Thailand’s geographical location within the Greater Mekong Subregion, sharing land borders with Cambodia, the Lao People’s Democratic Republic (Lao PDR), and Myanmar, offers opportunities for enhancing trade and investment through enhanced regional infrastructure and transportation development (Chirathivat , 2015). This results in the annual movement of millions of people and animals across diverse geographical areas and simultaneously increases the risk of infectious diseases and AMR dissemination. Therefore, understanding AMR in this region is critical for developing effective solutions on a global scale.

Nontyphoidal *Salmonella* (NTS) is a major foodborne pathogen primarily causing diseases in humans and animals. More than 2,500 serovars are classified as NTS, of which *Salmonella* Enteritidis is among the most prevalent serovars globally and is typically associated with poultry and eggs (Hull et al., 2022). While pig and pork are less common sources, the presence of *Salmonella* Enteritidis in the pig production chain remains an important concern for both food safety and AMR. *Salmonella* infection has become increasingly challenging due to the rapid emergence and spread of *Salmonella* strains resistant to clinically important antimicrobial agents (WHO, 2018). From a public health perspective, *Salmonella* is recognized as a key zoonotic agent recommended for inclusion in AMR surveillance programs (EFSA et al., 2019). MDR Salmonellosis cases have been on the rise globally (WHO, 2018). Although it is typically self-limiting, the pathogen has acquired various defense mechanisms to overcome host physical barriers and employs virulence factors to evade the host’s immune system. *Salmonella* often carries transmissible resistance (R) plasmids harboring multiple AMR genes, which contribute to its resistance against clinically important antibiotics and facilitate the spread of AMR. A recent study demonstrated the presence of various plasmids distributed in pigs, pork, and humans as well as the strong association between plasmids and various resistance phenotypes (Puangseree et al., 2022). Besides AMR genes, *Salmonella* also carries plasmid associated virulence genes (*e.g.*, *spvC, pefA,* and *rck*) (Pilla & Tang, 2018). The *spvC* gene is a *Salmonella* virulence plasmid-borne gene within the *spv* operon, playing a key role the invasion and systemic infection (Pilla & Tang, 2018). The *pefA* gene encodes plasmid-encoded fimbriae, contributing to the adhesion to epithelial cells. The *rck* gene encodes a protein that confer resistance to complement killing protein, contributing to the modulation of the host’s immune response (Pilla & Tang, 2018).

Virulence plasmids and R plasmid of different Inc groups may be colocalized in the same cell (*e.g.*, IncX1 and IncA/C2 identified in hybrid plasmid of *Salmonella* Dublin (Mangat et al., 2017)) or IncF, Incl1 and IncX1 in the same *Salmonella* Enteritidis (Liu et al., 2024). Simultaneously, coexistence of AMR and virulence genes on the same plasmids has been identified in several bacterial pathogens (Jain et al., 2018), including *Salmonella* serovars, for example, *Salmonella* Typhimurium (Guerra et al., 2002), *Salmonella* Enteritidis (García et al., 2016), *Salmonella* Dublin (Mangat et al., 2017), *Salmonella* Choleraesuis (Chen et al., 2020) and *Salmonella* Infantis (Bogomazova et al., 2020). These highlight the potential for a single antimicrobial drug to simultaneously select for both virulence and AMR genes, favoring the development of more severe infections. Virulence plasmids containing AMR genes potentially originated from the recombination and translocation of both R plasmids and virulence plasmids, facilitated by mobile genetic elements (*e.g.*, insertion sequences, integrons) (García et al., 2016; Mangat et al., 2017). Examples include IS*26* transposition that caused the incorporation of many AMR genes into pSEVT creating pUO-SeVR1 a hybrid plasmid of *Salmonella* Enteritidis-human isolate (García et al., 2016) and pN13-01125, a hybrid plasmid in *Salmonella* Dublin, comprising pSDVr and pSH696_135 plasmids (Mangat et al., 2017).

Recognizing the significant role of plasmids in AMR distribution, plasmid-based tracking has been recommended for inclusion in AMR surveillance programs (Wheeler et al., 2023). At the same time, the frequency and patterns of virulence factors vary over time and are not usually tracked. Identifying potentially virulent strains could provide valuable information at different levels. At a large scale, it helps understanding their prevalence, transmission pathways, and evolutionary trends. Concurrently, understanding the dynamics of AMR and virulence requires knowledge of genetic components, evolutionary processes, and variations among strains at an individual level (Borah et al., 2022; Zakaria et al., 2021). It is important to note, however, that the presence of virulence-associated factors does not necessarily indicate a virulent phenotype, as their expression may be under regulatory mechanisms and influenced by environmental conditions. However, the lack of high-quality AMR data is common in many LMICs, leading to inadequate control strategy in many counties. Whole genome sequencing (WGS) analysis has been demonstrated to enhance the quality and insights of AMR data that facilitate identifying genetic variations among strains and better understanding the persistence and spread of resistance and virulence plasmids (Oniciuc et al., 2018). This comprehensive approach offers valuable information for tracking the evolution and managing the dissemination of *Salmonella* strains (Mangat et al., 2017). Therefore, this study aims to investigate phenotypic characteristics of *Salmonella* isolates from pigs, pork, and humans in Thailand and its neighboring countries, as well as genetic characteristics of *Salmonella* carrying virulence plasmids and coexisting with AMR genes using WGS analysis.

## Materials & Methods

### *Salmonella* isolates

A total of 366 *Salmonella* isolates were included in this study (Table 1). They were previously isolated during AMR monitoring studies and stored as our bacterial culture collection. These isolates were collected between 2010 and 2024 from provinces bordering Thailand (Khon Kaen, Chiang Mai, Chiang Rai, Maha Sarakham, Nong Khai, Roi Et, Sa Kaeo, and Ubon Ratchathani, *n* = 181), Lao PDR (Vientiane and Savannakhet, *n* = 100), Cambodia (Banteay Meanchey, *n* = 67), and Myanmar (Tachileik, *n* = 18). They were isolated from rectal swab of clinically healthy pigs (*n* = 265) at slaughterhouses after bleeding but before scalding process, retail raw meat (*n* = 69) collected from fresh markets, human stool samples (*n* = 31) collected from diarrheal patients (*n* = 11) at local hospitals, slaughterhouse workers (*n* = 19) and butchers in fresh markets (*n* = 2). All were isolated using ISO 6579:2017 (ISO, 2017), serotyped using slide agglutination test (Grimont & Weill, 2007) and stored as 20% glycerol in our −80 °C bacterial stock. One colony of each serovars was collected from each positive sample. Research protocols involving human subjects were approved by the Ethics Committee of the Faculty of Medicine of Khon Kaen University with the authorization ID, HE572136. Self-collected stools or rectal swabs and informed consent forms were received from all participants.

**Table 1:** Distribution of isolates from pigs, pork and human across Thailand, Lao PDR, Cambodia and Myanmar (*n* = 366).

**Sample source**	**Sample type**	**Sampling location**	**Country**	**No. of isolates**
Pigs (*n* = 265)	Rectal swab	Slaughterhouse	Thailand	110
			Lao PDR	88
			Cambodia	67
Pork (*n* = 69)	Pork meat	Fresh market	Thailand	51
			Myanmar	18
Humans (*n* = 32)	Patient stool	Hospital	Thailand	11
	Butcher stool	Fresh market	Thailand	2
	Worker stool	Slaughterhouse	Thailand	7
			Lao PDR	12
**Total**				366

### Antimicrobial susceptibility testing

Minimum inhibitory concentration (MIC) values of all *Salmonella* isolates were examined by broth microdilution method using Sensititre™ Complete Automated AST System (Thermo Fisher, Waltham, MA, USA). The regionally customized Asia surveillance plates, ASSECAF and ASSECB, were used (TREK Diagnostic Systems, West Sussex, UK), of which antimicrobial agents included (abbreviations and clinical breakpoints in parentheses) ampicillin (AMP, 32 µg/mL), azithromycin (AZI, 32 µg/mL), chloramphenicol (CHL, 32 µg/mL), colistin (COL, 2 µg/mL), ciprofloxacin (CIP, 4 µg/mL), cefotaxime (FOT, 4 µg/mL), gentamicin (GEN, 16 µ/mL), meropenem (MERO, 4 µg/mL), nalidixic acid (NAL, 32 µg/mL), streptomycin (STR, 32 µg/mL), sulfamethoxazole (SMX, 512 µg/mL), ceftazidime (TAZ, 16 µg/mL), trimethoprim (TMP, 16 µg/mL), tetracycline (TET, 16 µg/mL) and tigecycline (TGC, 1 µg/mL) (CLSI, 2013). *Escherichia coli* ATCC 25922, *Pseudomonas aeruginosa* ATCC27853 and *Staphylococcus aureus* ATCC 29213 served as quality control strains. Multidrug resistance (MDR) was defined when the isolates were resistant to three or more antimicrobials of different classes (Magiorakos et al., 2012).

### PCR amplification of plasmid-encoded virulence genes

For all PCR assays, whole cell boiling DNA templates were prepared as previously described (Chaudhary et al., 2015). PCR assays were carried out using Toptaq Master Mix kit (Qiagen, Hilden, Germany) according to the manufacturer’s instructions. Three plasmid-associated virulence genes were screened in all *Salmonella* isolates (*n* = 366) using specific primer pairs as follows: *spvC* (spvC-F, ACTCCTTGCACAACCAAATGCGGA/ spvC-R, TGTCTTCTGCATTTCGCCACCATCA) (Chiu & Ou, 1996), *pefA* (pefA-F, GCGCCGCTCAGCCGAACCAG/ pefA-R, GCAGCAGAAGCCCAGGAAACAGTG) (Skyberg, Logue & Nolan, 2006) and *rck* (rck-F, TCGTTCTGTCCTCACTGC/rek-R, TCATAGCCCAGATCGATG) (Guerra et al., 2002). An isolate of *Salmonella* Enteritidis isolated from chicken served as the positive control for all three virulence genes (Sinwat, Angkittitrakul & Chuanchuen, 2015). PCR products were purified using Nucleospin^®^ Gel and PCR clean up (Macherey–Nagel, Düren, Germany) and submitted for DNA sequencing at First Base Laboratories (Selangor Darul Ehsan, Malaysia). Nucleotide sequences were compared with those available at GenBank database using the Blast algorithm (http://www.ncbi.nlm.nih.gov).

### Whole genome sequencing analysis

The five *Salmonella* isolates simultaneously carrying three plasmid-associated virulence genes (*i.e., spvC*, *pefA*, and *rck*) were analyzed using WGS (*n* = 5). Genomic DNA was purified using ZymoBIOMICS™ DNA Miniprep Kit (Zymo Research Corp., Irvine, CA, USA) following the manufacturer’s instructions. The integrity of the genomic DNA was assessed by running 5 µL of the DNA on 0.8% agarose gel stained with RedSafe™ nucleic acid staining solution (Thermo Fisher Scientific, Waltham, MA, USA). The concentration and quality of the extracted DNA were measured using a NanoDrop™ 1000 spectrophotometer (Thermo Fisher Scientific, Waltham, MA, USA) and submitted for WGS using Oxford Nanopore technologies (ONT) for long read sequencing at Siriraj Long-read Lab, Faculty of Medicine Siriraj Hospital, Mahidol University, Bangkok, Thailand and using Illumina platform Hiseq sequencers (Illumina, San Diego, CA, USA) for short read sequencing at GENEWIZ China and Suzhou Lab (GENEWIZ, Suzhou, China).

Genomic sequencing analysis was conducted as previously described (Arigul et al., 2023). Briefly, adapters were trimmed using Porechop v0.2.4 (https://github.com/rrwick/Porechop). ONT and Illumina reads were quality checked using NanoPlot (De Coster et al., 2018) and FastQC (Andrews, 2010), respectively. High quality ONT and Illumina reads were assembled to create hybrid genome using Unicycler (Wick et al., 2017). Genomic characteristics including genome size, number of contigs and % GC content were identified using QUAST (Gurevich et al., 2013). Taxonomic identification was performed using Kraken2 (Wood, Lu & Langmead, 2019). Genomic sequences were extracted utilizing Geneious Prime software (Biomatters, Ltd., Auckland, New Zealand). Genome annotation was performed using the Prokka software (Seemann, 2014) and NCBI Prokaryotic Genome Annotation Pipeline (PGAP) (Sayers et al., 2023). Identification of plasmid-associated virulence genes were achieved through the Virulence Factor Database (VFDB) available at http://www.mgc.ac.cn/VFs/main.htm (Liu et al., 2022). The assembled genome/contigs were further analyzed at Center for Genomic Epidemiology website (http://www.genomicepidemiology.org/services/). The prediction of AMR genes was conducted using ResFinder (Zankari et al., 2012) together with Resistance Gene Identifier (RGI) of the Comprehensive Antibiotic Resistance Database (CARD) (Alcock et al., 2023). *Salmonella* Pathogenicity Islands (SPIs) were analyzed using *Salmonella* Pathogenicity Islands Finder version 2.0 (SPIFinder 2.0) (Roer et al., 2016).

Mobile genetic elements (MGE) and plasmids were identified by MobileElementFinder v1.0.3 (Johansson et al., 2021) and PlasmidFinder2.1 (Camacho et al., 2009; Carattoli et al., 2014b), respectively. Multi-Locus Sequence Typing (MLST) (Larsen et al., 2012) and plasmid MLST (pMLST) (Carattoli et al., 2014a) were carried out. Plasmid incompatibility groups were also determined. The Proksee web-based platform was used to provide the visual representation of the plasmids obtained. Gene clusters from virulence plasmids and reference plasmid were aligned and comparatively analyzed using Clinker (Gilchrist & Chooi, 2021).

The default values on all parameters were used in all searches. The pSLT virulence plasmid of *Salmonella* Typhimurium str. LT2 (accession number AE006471.2) was used as the reference plasmid. Variant calling and core genome alignment was performed by Snippy (Seemann, 2015) and the phylogenetic trees were generated by IQ-TREE (Nguyen et al., 2015) and visualized by iTOL v6 (Letunic & Bork, 2021). Plasmids used for phylogenetic tree construction included pSLT, pSCV50 of *Salmonella* Choleraesuis SC-B67 (accession number: AY509003.1) and PCT02021853_74 of *Salmonella* Dublin CT_02021853 (accession number: CP001143.1). The whole genome sequences of SA607, SA608, SA615, SA616, and SA617 were deposited in National Center for Biotechnology Information (NCBI) under the accession numbers SAMN37159800, SAMN37160125, SAMN37156895, SAMN37156981 and SAMN37156982, respectively.

### Conjugation experiment

Biparental mating technique was performed using *Salmonella* isolates carrying at least one virulence gene (*n* = 5) as a donor strains and rifampicin-resistant derivatives of *E. coli* K12 strain MG1655 (MG1655Rif^r^) (rifampicin MIC = 256 µg/mL) was used as recipient strains. Transconjugants were selected on LB agar (Difco, BD Diagnostic Systems, Maryland, USA) containing 32 µg/mL of rifampicin and 100 µg/mL of ampicillin. The presence of corresponding plasmid-associated virulence genes in transconjugants were detected by PCR with specific primers as described above.

## Results

### *Salmonella* serovar

Overall, *Salmonella* Rissen was the most frequent serotype (29.51%), followed by *Salmonella* Typhimurium (12.57%) (Table 2). Serovar Rissen was also predominant in the pig (11.48%), and pork (9.02%) isolates from Thailand and the pig isolates from Cambodia (5.74%). The most common serovar among pig isolates from Lao PDR was Stanley (5.19%), while in Myanmar, the most frequent serovar in pork was Anatum (3.83%). Most of the human isolates were *Salmonella* Enteritidis (21.88%), followed by *Salmonella* Typhimurium (12.50%). The predominant serovar among Thai patients was Enteritidis (7/11). The human isolates from Lao PDR displayed a variety of serovars, including Weltevreden (*n* = 2), and one isolate each of Anatum, Derby, Give, Lexington, London, Rissen, Singapore, Stanley, Typhimurium, and Vejle.

**Table 2:** Common *Salmonella***serovar detected in this study** (*n* = 366).

***Salmonella*** ** Serovar**	Pigs (*n* = 265)			**Pork (*n* = 69)**		**Human (*n* = 32)**		**Total**
	**Thailand**	**Lao PDR**	**Cambodia**	**Thailand**	**Myanmar**	**Thailand**	**Lao PDR**	
Anatum	8 (3.01%)	14 (5.28%)	1 (0.38%)	2 (2.90%)	14 (20.29%)		1 (3.12%)	40 (10.93%)
Brunei		5 (1.89%)						5 (1.37%)
Derby	2 (0.75%)	4 (1.51%)	6 (2.26%)				1 (3.12%)	13 (3.55%)
Enteritidis						7 (21.87%)		7 (1.91%)
Hvittingfoss			1 (0.38%)	3 (4.35%)		2 (6.25%)		6 (1.64%)
Rissen	42 (15.85%)	7 (2.64%)	21 (7.92%)	33 (47.83%)	2 (2.90%)	2 (6.25%)	1 (3.12%)	108 (29.51%)
Sanktmarx	5 (1.89%)	1 (0.38%)						6 (1.64%)
Sao	12 (4.53%)							12 (3.28%)
Stanley	2 (0.75%)	19 (7.17%)	3 (1.13%)			1 (3.12%)	1 (3.12%)	26 (7.10%)
Typhimurium	21 (7.92%)	15 (5.66%)	5 (1.89%)	1 (1.45%)		3 (9.37%)	1 (3.12%)	46 (12.57%)
Weltreverden	6 (2.26%)	5 (1.89%)	2 (0.75%)			1 (3.12%)	2 (6.25%)	16 (4.37%)
Others	12 (10.91%)	18 (20.45%)	28 (41.79%)	12 (23.53%)	2 (11.11%)	4 (20.00%)	5 (41.67%)	81 (22.13%)

### Antimicrobial susceptibilities

**Figure 1:**
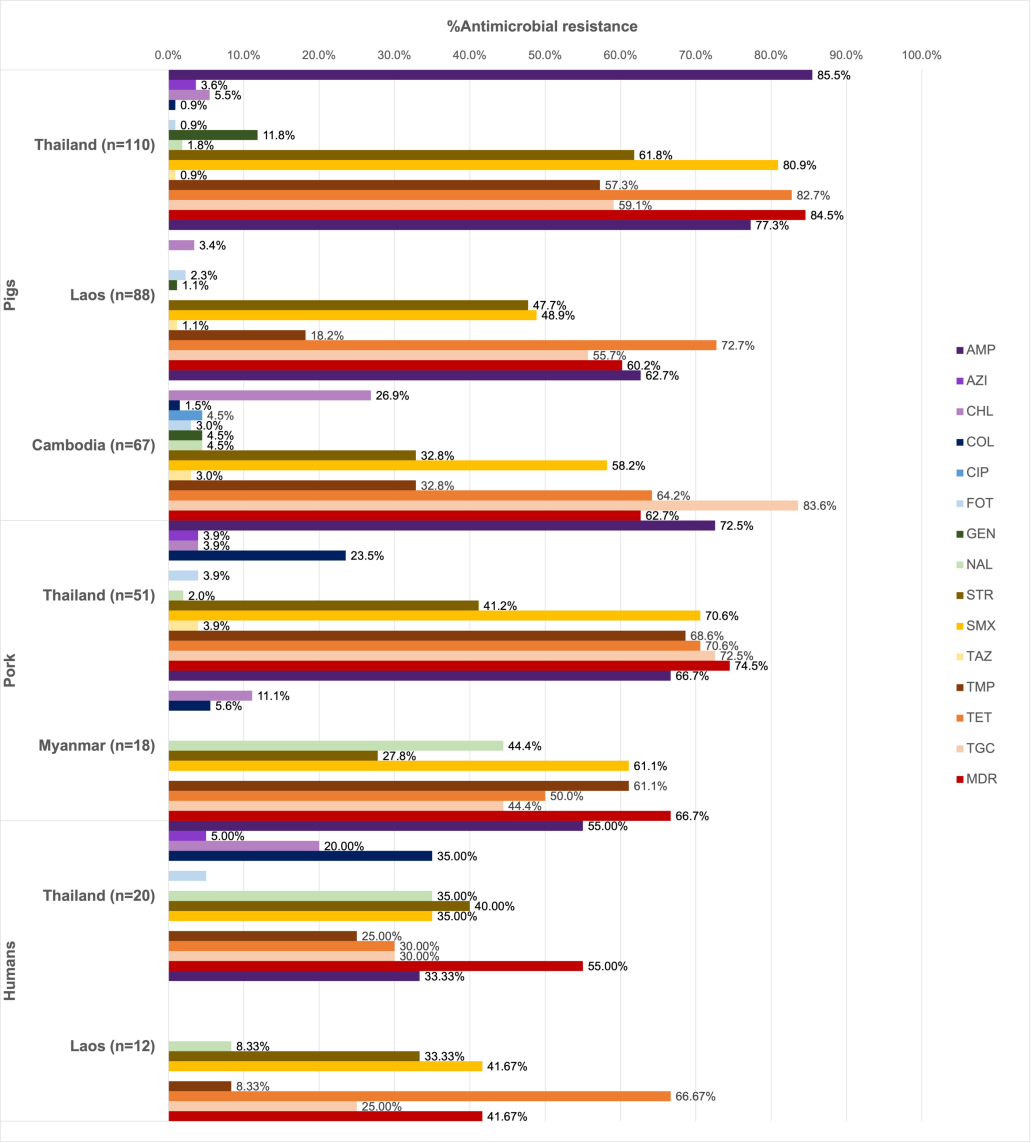
Antimicrobial resistance rates of pig, pork and human isolates (*n* = 366). Resistance rates of different antimicrobials are illustrated in distinct colors. AMP, ampicillin; AZI, azithromycin; CHL, chloramphenicol; COL, colistin; CIP, ciprofloxacin; FOT, cefotaxime; GEN, gentamicin; MERO, meropenem; NAL, nalidixic acid; STR, streptomycin; SMX, sulfamethoxazole. TAZ, ceftazidime; TMP, trimethoprim; TET, tetracycline; TGC, tigecycline.

Of all the isolates tested (*n* = 366), 90.16% were resistant at least one antimicrobial agent, and MDR was common (69.40%). High resistance rates to ampicillin (73.22%), tetracycline (70.22%), and sulfamethoxazole (62.84%) were observed. Ciprofloxacin resistance was observed only among the pig isolates from Thailand (1.13%), while all isolates remained susceptible to meropenem (Fig. 1).

Among pig isolates (*n* = 265), the highest resistance to ampicillin was observed in Thailand (85.45%) and Lao PDR (77.27%), while the highest resistance to tigecycline was reported in Cambodia (83.58%). Resistance to cefotaxime (1.89%) and ceftazidime (1.51%) was detected across the three countries. Colistin resistance (0.75%) was identified in both Thailand and Cambodia, whereas resistance to azithromycin (1.51%) was found solely in the Thai isolates.

On the other hand, high tigecycline resistance rate (72.55%) was detected in the pork isolates from Thailand. Colistin resistance (18.84%) was observed in isolates from Thailand and Myanmar, while resistance to azithromycin (2.90%), cefotaxime (2.90%), and ceftazidime (2.90%) was identified exclusively in Thailand.

In addition to ampicillin, the human isolates from Thailand (*n* = 20) exhibited resistance to streptomycin (40.00%), nalidixic acid, colistin, and sulfamethoxazole (35.00% each). Among these, those from Thai worker in slaugtherhouses (*n* = 7) were additionally resistant to tigecycline (71.43%, 5/7). Resistance to azithromycin (3.13%), colistin (21.88%) and cefotaxime (3.13%) were observed in the patient isolates (*n* = 11). For Lao PDR, all the isolates were from slaughterhouse workers (*n* = 12) and showed common resistance to tetracycline (66.67%, 8/12) and sulfamethoxazole (41.67%). Besides ciprofloxacin, gentamicin, and ceftazidime resistance was not detected.

A total of 67 AMR patterns were identified (Table 3), with AMP-SMX-TMP-TET-TGC being the most predominant (12.02%). The most common patterns varied by source: AMP-STR-SMX-TMP-TET-TGC in pigs (9.43%), AMP-SMX-TMP-TET-TGC in pork (27.54%), and COL-NAL in humans (12.50%, 4/32).

**Table 3:** The most common antimicrobial resistance patterns of ***S.enterica*isolates in this study** (*n* = 366 ).

**The most common** ** antimicrobial resistance pattern**	**Number of isolates (%)**	**Serovar (no.)**		
		**Pigs**	**Pork**	**Human**
AMP-SMX-TMP-TET-TGC	44 (12.02%)	Rissen (19), Anatum (3), Derby (1), Sao (1)	Rissen (15), Anatum (3), Norwich (1)	Rissen (1)
AMP-STR-SMX-TMP-TET-TGC	38 (10.38%)	Rissen (12), Anatum (5), Sanktmark (3), Braenderup (1), Sao (2), Typhimurium (1), Warragul (1)	Rissen (12)	Rissen (1)
AMP-STR-SMX-TET-TGC	25 (6.83%)	Typhimurium (13), Anatum (4), Stanley (2), Saintpaul (1), Sanktmark (1), Sao (1), Tsevie (1), Vilvoorde (1)		Vejle (1)
TGC	24 (6.56%)	Derby (2), Newmexico (2), Parathyphi (2), Stanley (2), Weltreverden (2), Bracknell (1), Corvallis (1), Dallgow (1), Dessau (1), Idikan (1), Koenigstuhl (1), Kouka (1), Parathyphi B (1), Stanley ville (1), Typhimurium (1), Yoruba (1)	Wien (1)	Weltreverden (1), Bardo (1)
AMP-STR-SMX-TET	22 (6.01%)	Typhimurium (19), Anatum (1), Augustenberg (1)		Typhimurium (1)
AMP-TET	16 (4.37%)	Stanley (7), Potto (3), Schwarzengrund (2), Kisii (1), Tsevie (1), Typhimurium (1)		Stanley (1)
AMP-GEN-STR-SMX-TMP-TET-TGC	12 (3.28%)	Rissen (12)		
AMP-STR-TET-TGC	11 (3.01%)	Stanley (3), Afulu (2), Augustenberg (1), Eingedi (1), Rissen (1), Saintpaul (1)	Rissen (2)	
AMP-CHL-STR-SMX-TMP-TET-TGC	10 (2.73%)	Derby (4), Rissen (3), Bradford (1), Panama (1), Tsevie (1)		

**Notes.**

AMP ampicillin AZI azithromycin CHL chloramphenicol COL colistin CIP ciprofloxacin FOT cefotaxime GEN gentamicin MERO meropenam NAL nalidixic acid STR streptomycin SMX sulfamethoxazole TAZ ceftazidime TMP trimethoprim TET tetracycline TGC tigecycline

### The presence of plasmid-associated virulence genes

Five strains (1.37%) of *Salmonella* Enteritidis (SA607, SA608, SA615, SA616, and SA617) were the only isolates that simultaneously carried *spvC*, *pefA*, and *rck* genes as detected by PCR. All were obtained from diarrheal patients in hospitals in Thailand (Table 4).

**Table 4:** Characteristics of *Salmonella* harboring plasmid-associated virulence genes (*n* =5).

**Isolates**	**Province**	**Resistanc phenotype**	**ST**	**Genome size (bp)**	**% GC content**	**Total** ** gene**	**Coding gene**	**Chromosome/ Plasmid**	**Plasmid replicon**	**Virulence genes**	**Resistance gene** ^a^	**Accession no.**
SA607	Chiang Mai	COL - NAL	11	4,777,006	52	4,679	4,382	Chromosome	–	–	*aac(6′)-Iaa, gyrA* mutation (G259T)	CP133435.1
								Virulence plasmid	IncFIB/ IncFII	*spvBCD, pefABCD, rck, mig-5*	ND	CP133436.1
SA608	Chiang Mai	COL - NAL		4,776,774	52	4,678	4,381	Chromosome	–	ND	*aac(6′)-Iaa, gyrA* mutation (G259T)	CP133438.1
								Virulence plasmid	IncFIB/ IncFII	*spvBCD, pefABCD, rck, mig-5*	ND	CP133439.1
SA615	Khon Kaen	AMP - COL - NAL - STR		4,790,057	52	4,692	4,394	Chromosome	–	–	*aac(6′)-Iaa, gyrA* mutation (G259T)	CP133426.1
								Virulence plasmid	IncFIB/ IncFII	*spvBCD, pefABCD, rck, mig-5*	ND	CP133427.1
								Resistance plasmid	IncX1	ND	*bla*_TEM−135_	CP133428.1
SA616	Khon Kaen	AMP - COL - NAL		4,790,057	52	4,692	4,394	Chromosome	–	-	*aac(6′)-Iaa, gyrA* mutation (G259T)	CP133429.1
								Virulence plasmid	IncFIB/ IncFII	*spvBCD, pefABCD, rck, mig-5*	ND	CP133430.1
								Resistance plasmid	IncX1	ND	*bla*_TEM−135_	CP133431.1
SA617	Chiang Mai	AMP - COL - FOT - NAL		4,790,057	52	4,692	4,394	Chromosome	–	–	*aac(6′)-Iaa, gyrA* mutation (G259T)	NZ_ CP133432.1
								Virulence plasmid	IncFIB/ IncFII	*spvBCD, pefABCD, rck, mig-5*	ND	NZ_ CP133433.1
								Resistance plasmid	IncX1	ND	*bla*_TEM−135_	NZ_ CP133434.1

**Notes.**

ND, Not detected.

a Only resistance genes and *gyrA* mutations identified in both ResFinder and CARD databases are shown.

### Genomic characteristics of *Salmonella* carrying plasmid-associated virulence genes (*n* = 5)

#### Genomic profile of *Salmonella* carrying virulence plasmids

The genome sizes of SA607 and SA608 were 4,777,006 bp and 4,776,774 bp, respectively, with a GC content of 52%. Their chromosome sizes were 4,717,634 bp and 4,717,402 bp, respectively. The other three isolates-SA615, SA616, and SA617-had a genome size of 4,790,057 bp and a chromosome size of 4,685,645 bp. All isolates belonged to sequence type 11 (ST11) with 13 types of SPIs identified on the chromosome including C63PI, CS54, an unnamed SPI of *Salmonella* Enteritidis CMCC50041 (accession no. CP013097.1), and SPI-1, SPI-2, SPI-3, SPI-4, SPI-5, SPI-7, SPI-9, SPI-10, SPI-11, SPI-12, and SPI-14 (Table S1). Three plasmid incompatibility (Inc) groups were identified: IncFIB and IncFII, present in all five isolates, and IncX1, specifically identified in SA615, SA616, and SA617.

#### Resistance genotype and R plasmids

Several AMR genes and mutations were detected on the chromosome (Table 4 and Table S2). Examples included the components of multidrug efflux system in the Resistance-Nodulation-Cell Division family (*e.g.*, CRP, *rsmA*, *marA*, *acrB*, *golS*, *adeF, sdiA, acrB*), the Major Facilitator Superfamily (*e.g.*, *emrB*, *emrR*, *leuO*), the Multidrug and Toxic Compound Transporter (*e.g.*, *mdtK*) and the Small Multidrug Resistance family (*e.g.*, *kpnE*, *kpnF*). Target alterations were also observed in genes (*e.g.*, *soxS/soxR*, *vanG, gyrA*) and amino acid chains (*e.g.*, penicillin-bonding protein-3 (PBP3), MarR, GlpT, EF-Tu, UhpT). Mobile genetic elements (MGEs) were also identified (Table S3).

**Figure 2:**
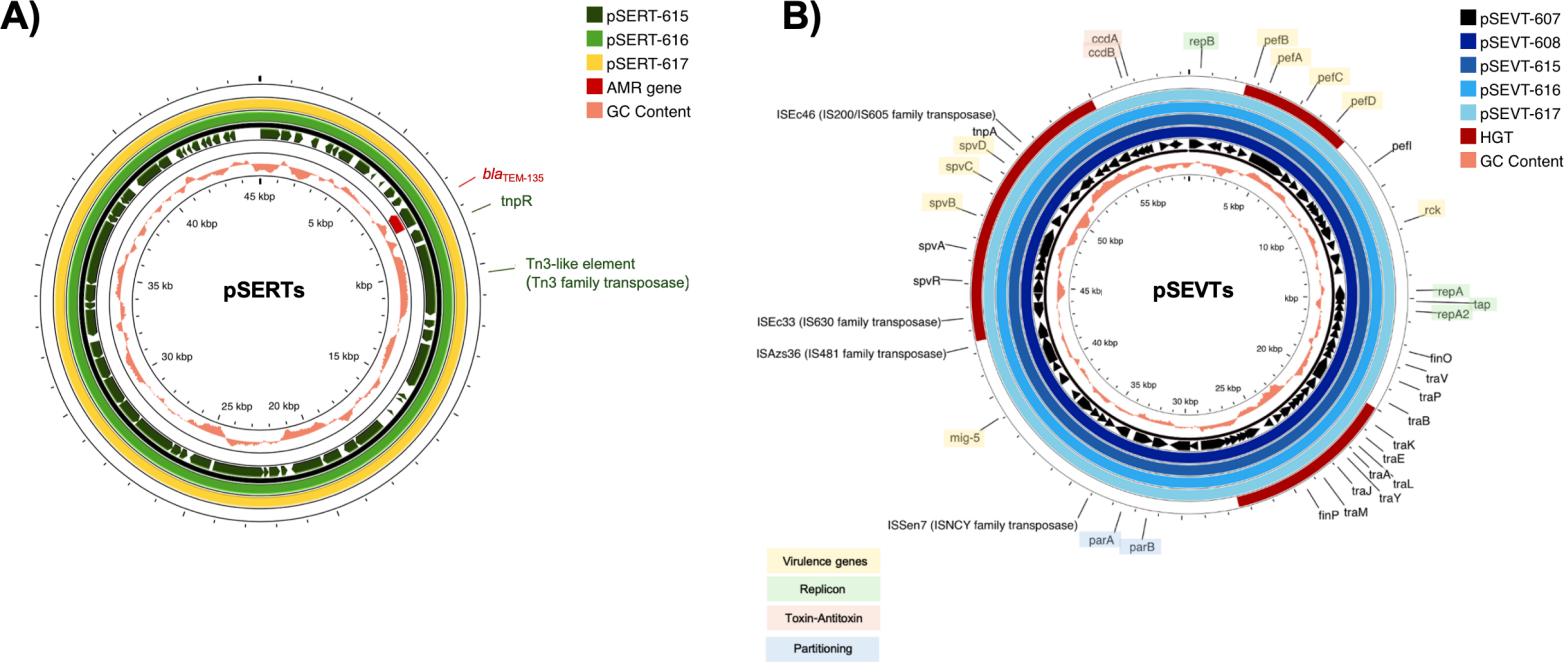
Circular alignment of R plasmid and virulence plasmid. (A) Comparison of R plasmids existing together with virulence plasmid named pSERT-615, pSERT-616 and pSERT-617. AMR genes are indicated in red text, and MGEs found on the same plasmid are also shown. (B) Comparison of *S. Enteritidis* virulence plasmids pSEVT-607, pSEVT-608, pSEVT-615, pSEVT-616, and pSEVT-617 from this study, arranged from the innermost to the outermost rings. The figure highlights virulence genes and essential genes of virulence plasmid survival and dissemination.

All isolates carried the chromosomally encoded *aac(6′)-Iaa* gene and G259→T point mutation in the quinolone resistance-determining region (QRDR) of *gyrA* leading to an amino acid substitution from aspartic acid to tyrosine at position 87 (Asp87→Tyr) in GyrA. Consistently, all isolates were resistant to nalidixic acid. Three *Salmonella* isolates resistant to ampicillin (SA615, SA616 and SA617) contained *bla*_TEM−135_ on IncX1 plasmid (Fig. 2A). A streptomycin-resistant isolate (SA615) and all five colistin-resistant isolates did not carry genes encoding resistance to corresponding antibiotics.

IncX1 R plasmids of 45,040 bp in size including pSERT-615, pSERT-616, and pSERT-617 were identified in SA615, SA616, and SA617, respectively. All plasmids showed 100% sequence identity. The plasmids carried AMR genes including *bla*_TEM−135_. Two MGEs identified on these plasmids included *tnpR* and *tn3*-like elements of Tn3 family of transposases (Fig. 2).

#### Genetic characteristics of virulence plasmids

Based on WGS analysis, all *Salmonella* Enteritidis tested PCR-positive for plasmid-associated virulence genes (*n* = 5) harbored *spvBCD*, *pefABCD*, *rck* and *mig-5* (macrophage-inducible gene-5) (Table 4).

All the IncFIB/IncFII plasmids, including pSEVT-607, pSEVT-608, pSEVT-615, pSEVT-616, and pSEVT-617, shared an identical size of 57,372 bp and exhibited 100% sequence identity (Table 4 and Fig. 2). All these virulence plasmids were classified as S1:A-:B22 based on pMLST analysis. None were found to carry AMR genes.

Nine plasmid-associated virulence genes were identified across all virulence plasmids. Each gene encodes a distinct type of virulence factor. The *spvB, spvC,* and *spvD* genes, components of the *spv* operon, span 6,047 bp and are located between nucleotide positions 44,672 and 50,719. The *pefB, pefA, pefC*, and *pefD* gene of the *pef* operon span 4,421 bp from nucleotide positions 2,612 to 7,033. The *rck* and *mig-5* genes cover 558 bp from nucleotide positions 11,474 to 12,031 and 741 bp from nucleotide 38,725 to 39,465, respectively. In addition to virulence genes, MGEs were identified on the virulence plasmid, including four insertion sequences - IS*Sen7* of the IS*NCY* family transposase, IS*Azs36* of the IS*481* family transposase, IS*Ec33* of the IS*630* family transposase and IS*Ec46* of the IS*200*/IS*605* family transposase - as well as *tnpA* a transposase gene located downstream of the *spv* operon (Fig. 2B).

**Figure 3:**
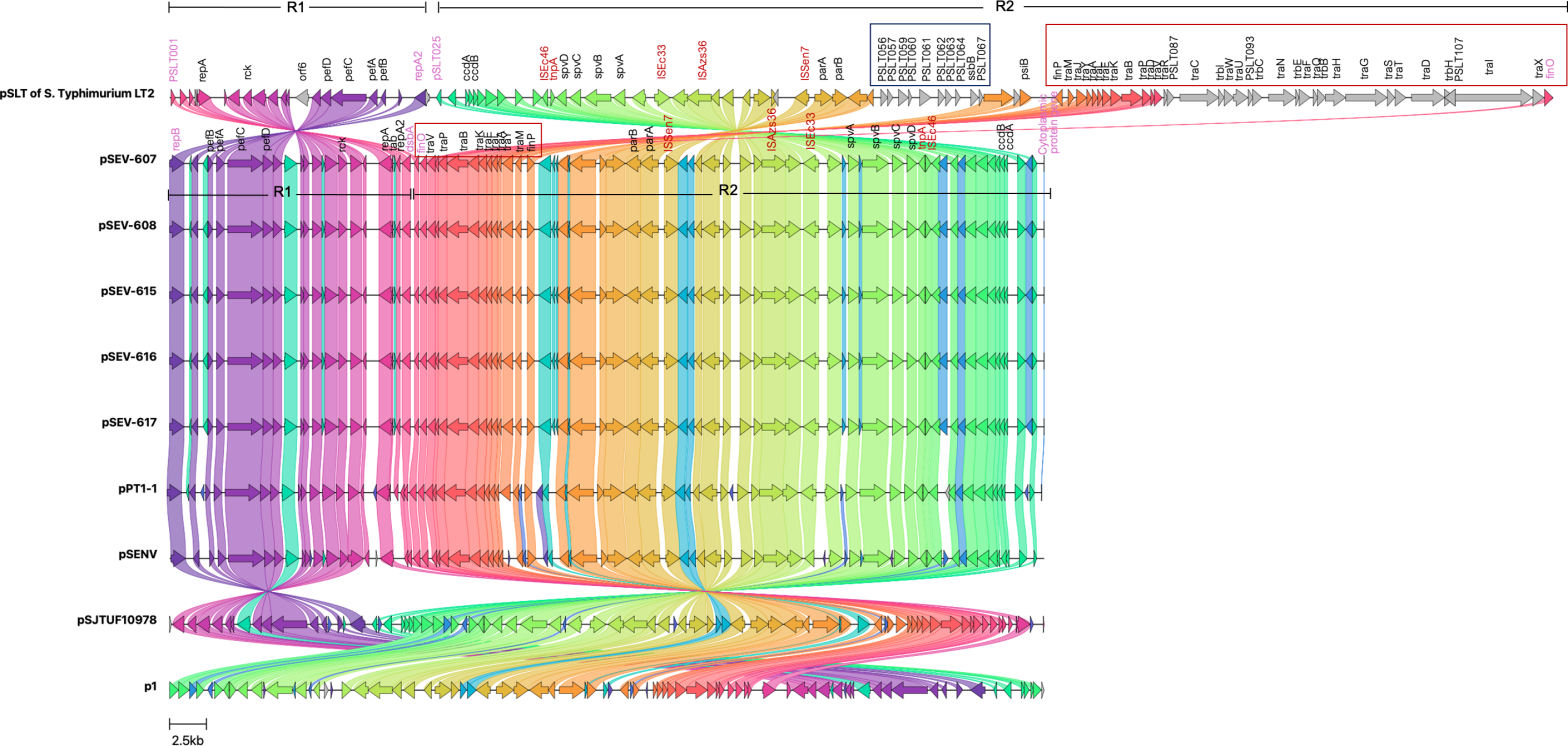
Genomic sequence alignment of the virulence plasmids in this study (pSEVT-607, pSEVT-608, pSEVT-615, pSEVT-616, and pSEVT-617) with the virulence plasmid pSLT of *S. Typhimurium* strain LT2 and highly identical plasmids from diverse regions. Homologous sequences are shown and linked in the same color. Grey arrows represent misalignments between plasmids. R1 and R2 are inverted homologous regions. The dark blue square highlights unique pSLT loci that are present in pSLT but absent in pSEVTs. The red square highlights variations in the *tra* operon. pSEVTs lack several genes in *tra* operon in pSLT. Highly identical plasmids of pSEVTs from diverse geographical regions were aligned for comparison including pSJTUF10978 from China (accession number: CP015525.1), p1 from South Korea (accession number: CP126167.1), pPT1-1 from the UK and pSENV from strain A1636 in Malawi.

Comparative genomic analysis revealed a high sequence identity between pSEVTs and pSLT (Fig. 3). The pSEVTs virulence plasmids exhibited high identity with plasmids of *Salmonella* Enteritidis from different geographical regions including pSJTUF10978 from China (accession number: CP015525.1) p1 from South Korea (accession number: CP126167.1), pPT1-1 from the UK (accession number: CP043434.1), and pSENV from strain A1636 in Malawi (accession number: CP063709.1).

Compared to the pSLT reference plasmid, all pSEVT plasmids exhibited a 99.4% identity. Since gene annotations were unavailable for some genes in pSLT, locus tags were used in place of gene names of these unidentified genes. The locus tags for the pSLT reference plasmid ranged from pSLT1 to pSLT111, while those for pSEVTs varied. Most regions of pSEVTs in this study were highly similar to those of pSLT, except for two segments that were absent in pSEVTs: PSLT056-PSLT057-PSLT059-PSLT060-PSLT061-PSLT062-PSLT063-064-ssB-PSLT067 and *traR-traC-trabI-traW-traU-trbC-traN-trbE-traF-traQ-traQ-trbB-traH-traD-traH-traI-traX* (Fig. 3). Two regions in pSLT, R1 spanning from locus tag PSLT001 to *repA2* and R2 spanning from PSLT025 to *finO*, were found to be inverted in pSEVTs. The R2 region additionally contained the deleted *tra* genes. The similar invert regions were found in pPT1-1 and pSENV, while the incomplete *tra* regions were also identified in pSJTUF10978, pPT1-1, pSENV and p1.

Five isolates of *Salmonella* harboring virulence plasmids were assessed for their ability to transfer plasmids under ampicillin selective pressure; however, no transconjugants were obtained under ampicillin selective pressure for plasmid transference.

### Genetic relatedness

**Figure 4:**
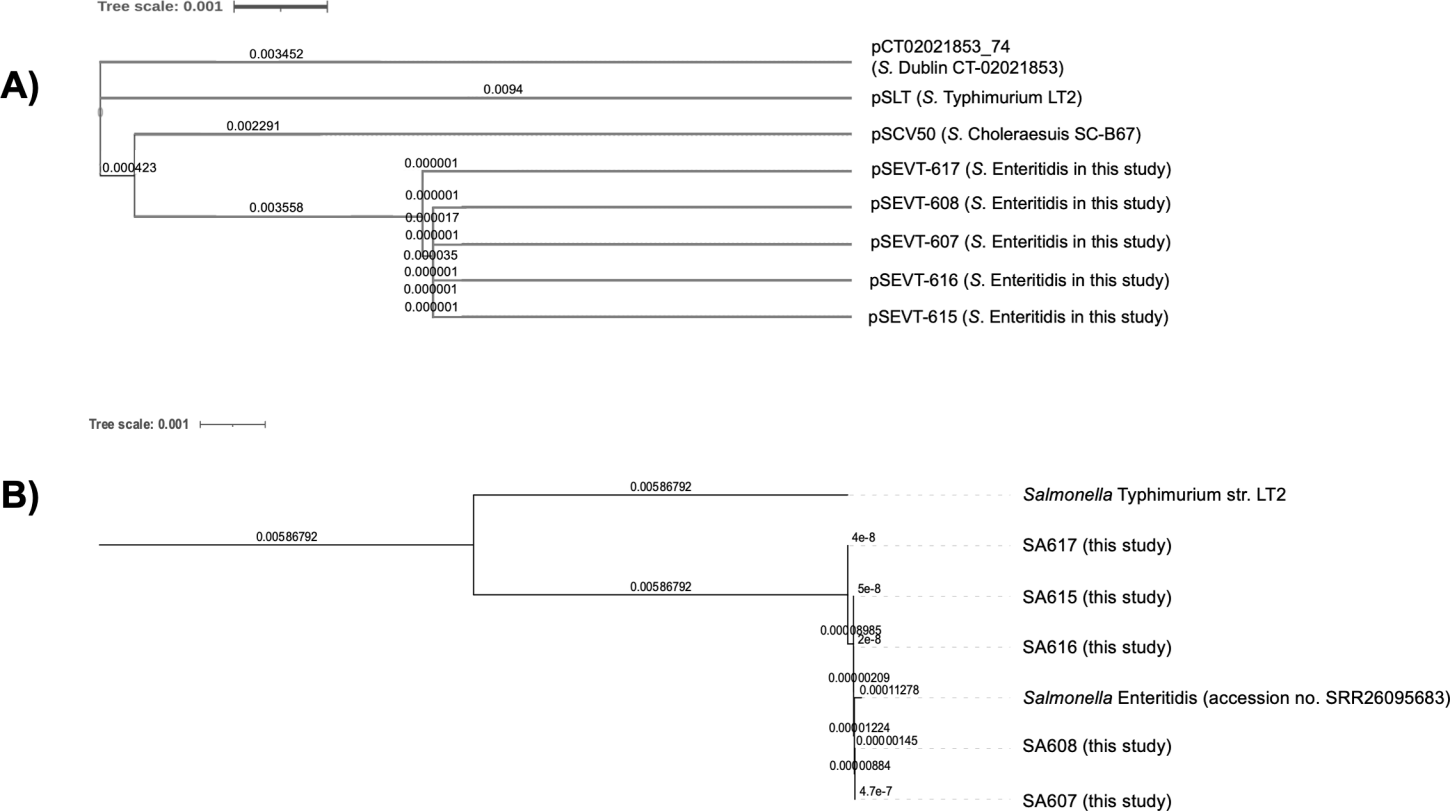
Phylogenetic tree of virulence plasmids from this study (*n* = 5). (A) This phylogenetic tree represents the genomic sequence alignment of virulence plasmids in this study (pSEVT-607, pSEVT-608, pSEVT-615, pSEVT-616, and pSEVT-617) compared with well-characterized virulence plasmids, pSLT from *S. Typhimurium* strain LT2, PCT02021853_T4 from *S. Dublin* strain CT_02021853, and pSCV50 from *S. Choleraesuis* strain SC-B67. (B) The genetic relatedness of SA607, SA608, SA615, SA616, and SA617 were analyzed based on their whole genome sequences. All the five *Salmonella* strains are in the same cluster and exhibited high genomic similarity to *Salmonella* Enteritidis (accession no. SRR26095683), indicating close genetic relatedness. In contrast, a genetic distinction from *Salmonella* Typhimurium LT2 was observed, demonstrating clear phylogenetic separation.

The phylogenetic analysis of virulence plasmids indicated a close genetic relationship among the five pSEVTs (Fig. 4). These plasmids were more closely related to each other than to virulence plasmids of *Salmonella* Typhimurium, pSCV50 of *Salmonella* Choleraesuis, and PCT02021853_74 of *Salmonella* Dublin. The phylogenetic tree of the core genome sequences was analyzed for SA607, SA608, SA615, SA616, and SA617. All the *Salmonella* strains are in the same clade and were similar to *Salmonella* Enteritidis (accession no. SRR26095683) isolated from raw chicken.

## Discussion

In this study, several *Salmonella* serovars were found, with serovar Rissen being the most prevalent among pig and pork isolates, aligning with regional trends in the pig industry (Lay et al., 2021; Seeharach et al., 2024). The frequent detection of *Salmonella* Enteritidis, *Salmonella* Typhimurium, and *Salmonella* Rissen in the human isolates from Thailand and Laos underscores their public health importance. These serovars are among the most commonly linked to potential zoonotic infections and the evidence of adaptation and persistence within pig production systems has been previously demonstrated for *Salmonella* Typhimurium and *Salmonella* Rissen (Campos et al., 2019). Although all *Salmonella* serovars can cause disease, certain serovars are particularly significant in terms of AMR and pose a greater threat for food safety (Arya et al., 2017; Elbediwi et al., 2021; Miller et al., 2010; Pornsukarom, Van Vliet & Thakur, 2018).

A key finding was the high MDR rates in isolates from clinically healthy pigs, pork, and humans in provinces bordering Thailand and land neighboring countries. Only healthy pigs are expected to be slaughtered for human consumption, but their health status does not ensure the absence of resistant bacteria, as antibiotics may have been applied previously. Given the dynamics of AMR, antibiotic use and AMR development may not always occur simultaneously. This is exemplified by the continued detection of chloramphenicol-resistant bacteria in the years following its ban in 1999 (Magiorakos et al., 2012), indicating that resistance can persist long after the antibiotic use has stopped. Additionally, pigs serve as asymptomatic carriers of AMR *Salmonella* that pose a major risk for the dissemination within pig production systems and transmission to humans through either derived food product or environment (Bonardi, 2017; Campos et al., 2019).

The observed AMR rates and patterns indicate concerning levels of resistance. The high resistance to ampicillin, tetracycline, sulfonamides, and trimethoprim is unsurprising, given their prolonged and widespread use in both veterinary and clinical settings (Caneschi et al., 2023; Kaushik et al., 2014). The high frequency of resistance to medically important antimicrobials is particularly alarming, such as tigecycline, which is approved exclusively for human use and reserved as a last-resort treatment for multidrug-resistant infections (Yaghoubi et al., 2022). The other examples include azithromycin resistance observed exclusively in Thai pig and pork isolates and ciprofloxacin resistance found only in Cambodian pig isolates. Both are considered key antimicrobial agents used to treat invasive *Salmonella* infections in humans (Gomes et al., 2017; Kariuki et al., 2015). Studies have shown that tigecycline resistance may result from tetracycline use (Linkevicius, Sandegren & Andersson, 2016). However, data on the use of these antibiotics in pig production and human healthcare in these countries remain limited.

The presence of *spvC*, *pefA*, and *rck* was limited to *Salmonella* Enteritidis clinical isolates from humans, underscoring their role in enhancing pathogenicity. This observation agrees with a previous study in China (Cheng, Eade & Wiedmann, 2019) and Thailand (Buddhasiri et al., 2023), supporting that the distribution of these genes is limited to certain serovars (Feng et al., 2012). At the same time, plasmid-associated virulence genes were absent in the pig and pork isolates is likely because they originate from clinically healthy pigs and pork meant for consumption, which aligns with a previous study on retail pork in Thailand (Patchanee et al., 2017) and pigs from slaughterhouse in the Philippines (Pavon et al., 2022). Plasmid-associated virulence genes have been reported more frequently in poultry compared to pigs, pork, or other food animals (Aung et al., 2022; Igbinosa et al., 2023). This may reflect the host-specific adaptation of virulence plasmidsto poultry, combining with high-density farming that promote horizontal plasmid transfer. Additionally, antimicrobial use and the shorter lifecycle of broilers further accelerates bacterial turnover and plasmid dissemination. Antimicrobial use and the short lifecycle of broilers further promote plasmid persistence and rapid dissemination (Farooq et al., 2022; Meng et al., 2024).

WGS identified all *Salmonella* Enteritidis isolates as belonging to ST11, a sequence type widely distributed in humans, animals, and animal-derived food (ECDC, 2023) and associated with salmonellosis outbreaks worldwide (Carroll et al., 2021; PhuHuong Lan et al., 2016). A previous study reported that the ST11 was predominantly found in human clinical isolates in Thailand (Buddhasiri et al., 2023) as well as in humans and chicken in China (Liu et al., 2024).

The chromosomally-encoded *aac(6′)-Iaa* gene was detected by WGS in all *Salmonella* Enteritidis clinical isolates (Table 4), in agreement with a previous study in the ST11 clinical isolates in Singapore and Senegal (Aung et al., 2022; Dieye et al., 2024) and in food and water samples in Russia (Kulikova et al., 2023). In contrast, it was absent in a recent study in China (Liu et al., 2024). This gene is regarded as a cryptic or conserved gene, with minimal to no functional contribution to phenotypic resistance to aminoglycosides in *Salmonella*; therefore, its presence alone should not be interpreted as indicative of resistance (Salipante & Hall, 2003). All isolates additionally harbored G259→T within QRDRs in *gyrA* contributing to nalidixic acid resistance, in agreement with previous studies (Aung et al., 2022; ECDC, 2022; Liu et al., 2024). Colistin is a last-resort antibiotic against MDR Gram-negative bacteria, especially carbapenem-resistant *Salmonella*. Plasmid mediated colistin resistance is primarily driven by the dissemination of *mcr*. However, all the isolates displayed colistin resistance without the presence of *mcr*, in agreement with a previous study in animals, the environment (Bertelloni et al., 2022) and humans (Fortini et al., 2022). These results suggest occurrence of *mcr*-independent colistin resistance mechanisms, *e.g.*, modifications to lipopolysaccharides, alterations in outer membrane synthesis and the activity of multidrug efflux pumps.

Two patterns of plasmid replicon, IncFIB-IncFII, and IncFIB-IncFII-IncX1, observed in this study are consistent with previous studies (Aung et al., 2022; Liu et al., 2024). A previous study showed that IncF replicons were common in *Salmonella* from pig, pork and humans and statistically associated with AMR in Thailand (Puangseree et al., 2024). The combination of IncFIB and IncFII plasmids has previously been shown to be uniquely linked to *Salmonella* virulence plasmids, particularly in *Salmonella* Typhimurium, *Salmonella* Enteritidis, and *Salmonella* Choleraesuis, which acquired these replicons through genetic evolution (Cheng, Eade & Wiedmann, 2019; Feng et al., 2012). IncX1 plasmids was found to be associated with hybrid plasmids containing multiple AMR genes and linked to MDR *Salmonella* Enteritidis ST11 (Cao et al., 2023). In this study, IncX1 plasmid in SA615, SA616 and SA617 only carried *bla*_TEM−135_, which encodes resistance to ampicillin and amoxicillin, consistent with their observed phenotypic profiles. Despite the limited number of isolates, the observation points out the increasing challenges in treating salmonellosis due to the coexistence of virulence and resistance plasmids within the same isolate.

Virulence plasmids typically range in size from 50 kb to 100 kb (Pilla & Tang, 2018), consistent with the current findings. To date, no reports have documented *Salmonella* virulence plasmids lacking *spvBCD* operon, suggesting the *spv* genes as a reliable indicator for screening *Salmonella* virulence plasmids (Tasmin, Gulig & Parveen, 2019). Besides, MGEs (IS*Sen7*, IS*Azs36*, ISEc33 and IS*Ec46* and *tnpA*) were present on these virulence plasmids. MGEs are recognized as critical drivers in shaping the structure and compositions of plasmids (García et al., 2016). The findings highlight the importance of monitoring MGEs on virulence plasmids as part of routine AMR surveillance to predict and prevent the emergence and spread of hybrid plasmids and high-risk bacterial clones (Cheng, Eade & Wiedmann, 2019), emphasizing the need to restrict antibiotic use and enforce rigorous infection control measures.

As a leading cause of zoonotic *Salmonella* infection worldwide, S*almonella* Enteritidis is of particular interest, especially for its virulence plasmids, which may enhance pathogenicity, drive evolution, and facilitate host adaptation. Notably, these plasmids frequently carry the *spv* operon and have been shown to facilitate intracellular survival and replication, in agreement with this study, thereby promoting systemic dissemination in infected hosts (Rychlik, Gregorova & Hradecka, 2006). The pSEVTs virulence plasmids showed a high degree of similarity to plasmids from *Salmonella* Enteritidis isolated in various geographical regions. The observations suggest that these plasmids may be evolutionarily conserved and potentially contribute to the enhanced pathogenicity and adaptability of *Salmonella* Enteritidis in varied environments (Chu et al., 1999). Since pSEVTs were detected in only a limited number of human isolates (1.37%), it is premature to suggest their widespread dissemination, however, underscoring the importance of monitoring plasmid-associated genes.

Despite the high similarity, pSEVTs contained the invert regions and incomplete *tra* operon in comparison to pSLT and pSJTUF10978 (Fig. 3). The underlying cause of these structural rearrangements remains unclear. However, they may represent evolutionary adaptations that contribute to plasmid stability and enhance adaptation to specific environmental or host conditions.

Based on our research, there are no reports of a similar inversion to date. A previous study showed the deleted *tra* operon in virulence plasmids of *Salmonella* Enteritidis (pSEV) and *Salmonella* Choleraesuis (pSCV) that was suggested to evolve from pSLT by partial deletion of the same operon (Cheng, Eade & Wiedmann, 2019). The presence of incomplete *tra* operon explained the observed lack of conjugative transfer capabilities, in agreement with a previous study (Cheng, Eade & Wiedmann, 2019; Feng et al., 2012). These studies suggested that the transmission of pSEV predominantly occurs *via* vertical transfer and is restricted to *Salmonella*.

Despite carrying *bla*_TEM−135_ gene, *in vitro* conjugative transfer was not observed for any of the pSERT plasmids. This may be due to the absence of key transfer elements in the plasmids or the need for specific conditions or helper plasmids not included in the *in vitro* assay. Further studies are needed to assess whether these plasmids can be mobilized by co-resident conjugative plasmids or transferred under different environmental conditions.

Phylogenetic analysis of virulence plasmids demonstrated a close relationship between the pSEVTs of *Salmonella* Enteritidis in this study, pSLT of *Salmonella* Typhimurium, and pSCV50 of *Salmonella* Choleraesuis SC-B67. This close genetic evolution within the pSLT lineage may be explained by the hypothesis that an ancestral IncFIIA plasmid merged with IncFIB to create pSLT, which then transferred the IncFIIA/IncFIB replicon to the virulence plasmids of *Salmonella* Enteritidis and *Salmonella* Choleraesuis (Cheng, Eade & Wiedmann, 2019). This was not the case for PCT02021853_T4 of *Salmnella* Dublin that evolved solely from the IncFIIA plasmid type and was far from others on phylogenetic tree (Cheng, Eade & Wiedmann, 2019). At the same time, phylogenetic analysis of whole genome sequences revealed that all the isolates had the same genetic evolution. These findings suggest the clonal dissemination of the isolates. Notably, all five *Salmonella* Enteritidis isolates collected from different patients and provinces (SA607/SA608/SA617-Chiang Mai and SA615/SA616-Khon Kaen) exhibited high genetic similarity in their pSEVTs, while three of them also carried pSERTs (SA615/SA616/SA617). The possible circulation of resistance plasmids among the isolates in this collection warrants further investigation. However, the sources of contamination remain unidentified due to the absence of patient history. It should be noted that the virulence plasmids appeared only in human isolates, with none detected in those from pigs or pork. This is not unexpected, as *Salmonella* Enteritidis is typically associated with poultry rather than swine. The observed lack of linkage between human and pig/pork isolates may therefore reflect source-specific serovar distribution, rather than a true absence of zoonotic transmission. Given the current sample composition, a conclusive assessment of zoonotic linkage cannot be made.

## Conclusions

In conclusion, the results emphasize the presence of *Salmonella* isolates from pigs, pork, and humans that exhibit resistance to multiple drugs, including last resort antibiotics. WGS has provided insights into the genetic components and structure of virulence and R plasmids in the *Salmonella* human isolates. Co-existence of virulence plasmids and R plasmids underscores the necessity of incorporating the identification of plasmid-associated virulence genes alongside AMR genes in AMR surveillance programs for both humans and animals. This more comprehensive approach could greatly improve the development of effective strategies for preventing and controlling resistant *Salmonella* infections.

##  Supplemental Information

10.7717/peerj.19884/supp-1 Supplemental Information 1 *Salmonella* Pathogenicity Islands (SPIs) identified in 5 *Salmonella* Enteritidis ST 11 in this study (SA607, SA608, SA615, SA616 and SA617)

10.7717/peerj.19884/supp-2 Supplemental Information 2 AMR determinants and mutations identified on chromosome of *Salmonella* Enteritidis ST 11 in this study (SA607, SA608, SA615, SA616 and SA617)

10.7717/peerj.19884/supp-3 Supplemental Information 3 Mobile genetic elements identified in 5 *Salmonella* Enteritidis, except Tn2 detected in only SA615, SA616 and SA617

10.7717/peerj.19884/supp-4 Supplemental Information 4 Raw data from WGS

## Additional Information and Declarations

### Field Study Permissions

The following information was supplied relating to field study approvals (*i.e.*, approving body and any reference numbers): Some Salmonella isolates were isolated from pork samples that were collected from the market in 2024. However, the permission to buy pork from the market is not required.

### DNA Deposition

The following information was supplied regarding the deposition of DNA sequences: Sequences are available at NCBI: CP133435.1, CP133436.1, CP133438.1, CP133439.1, CP133426.1, CP133427.1, CP133428.1, CP133429.1, CP133430.1, CP133431.1, CP133432.1, CP133433.1, CP133434.1.

### Data Availability

The following information was supplied regarding data availability: Raw data is available in the Supplemental Files.
